# Supplementary material for: Rational design of DNA nanostructures for single molecule biosensing
Source: Nat Commun. 2020 Sep 1;11:4384. doi: 10.1038/s41467-020-18132-1 (PMC7463249; doi:10.1038/s41467-020-18132-1)
Supplement: Supplementary file 1 — Supplementary Information [file 41467_2020_18132_MOESM1_ESM.pdf]

Rational design of DNA nanostructures for single molecule biosensing

Raveendran et al.

Supplementary Information

Supplementary Figures **2**

Supplementary Tables **21**

Supplementary Figures

Concentric squares translocation peak characterisation.

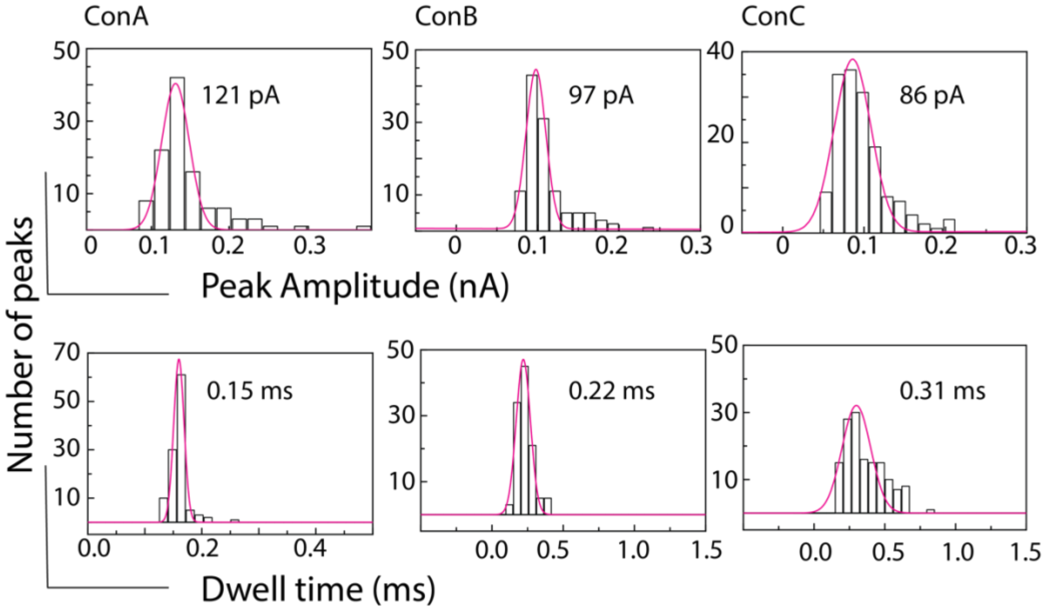

Supplementary Figure 1: Peak amplitude and dwell time histograms of ConA, B and C. The mean values are stated in the figure.

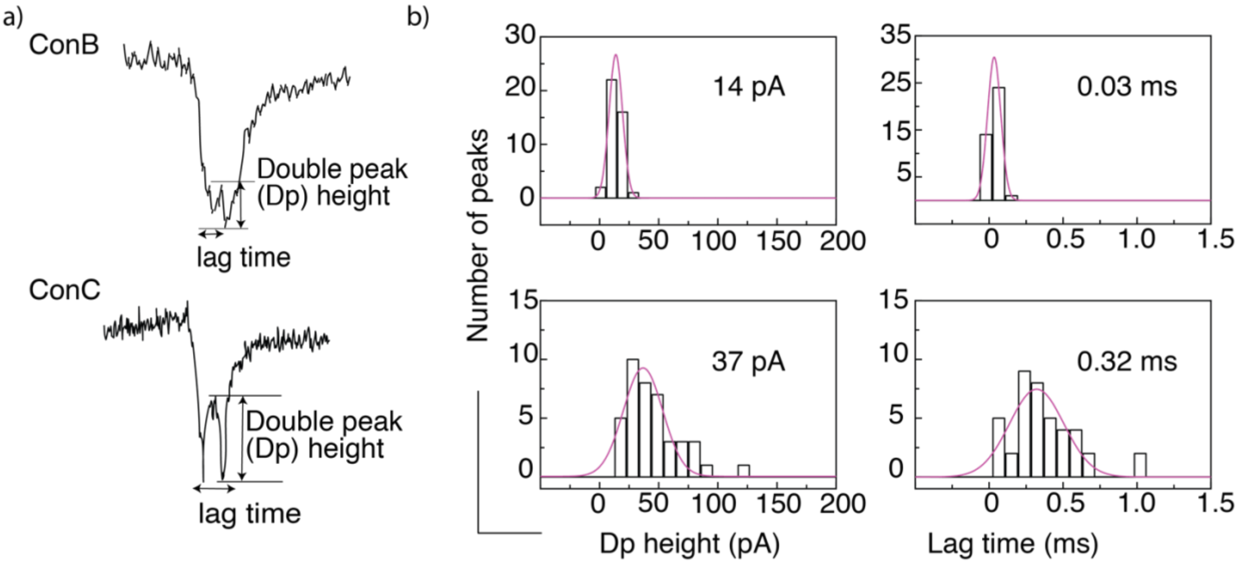

Supplementary Figure 2: Lag time and double-peak (Dp) height analysis for the two concentric square nanostructures featuring central cavities (ConB and ConC). The mean values are stated in the figure.

DNA aptamer characterisation

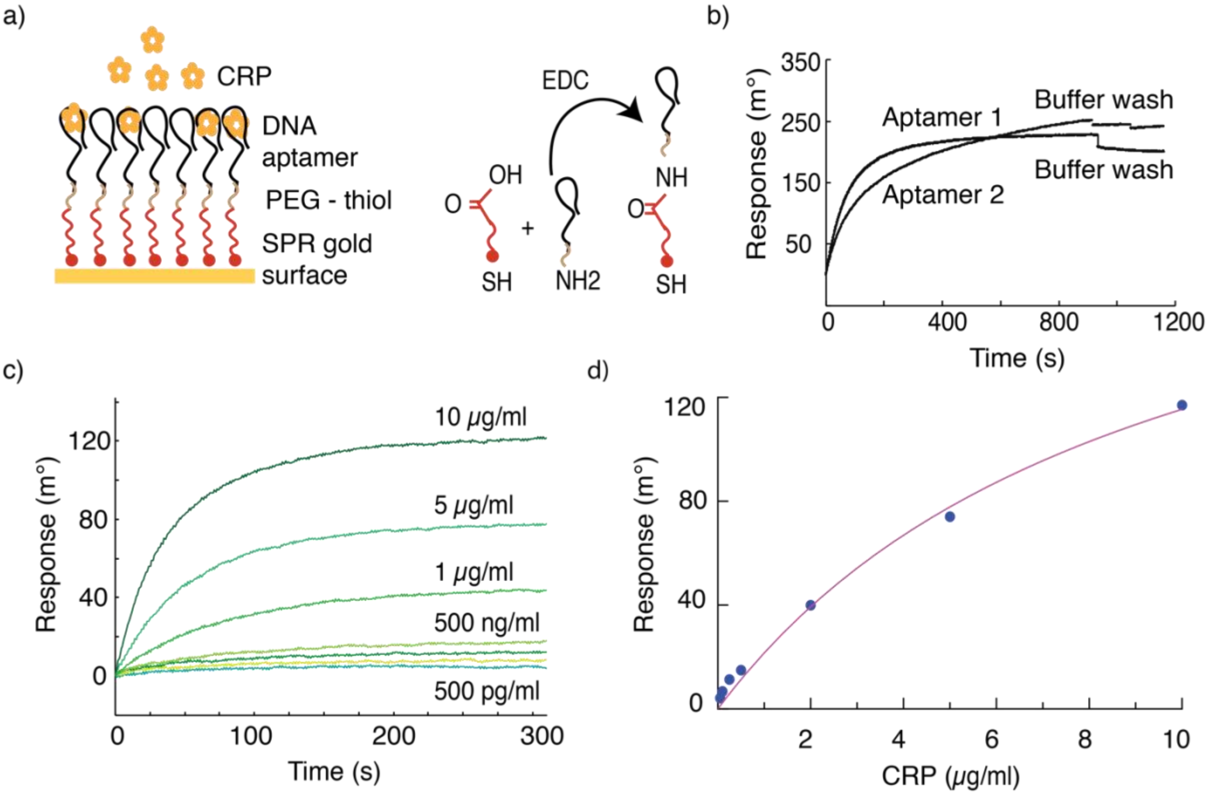

Supplementary Figure 3: SPR studies for CRP aptamer selection: a) Schematic of DNA aptamer attachment to SPR sensor surface. A mercapto-PEG-COOH was formed on the SPR gold surface for the attachment of amine-modified DNA aptamers via EDC. b) The SPR sensogram for the two DNA aptamers shows that they both bind CRP and hence are suitable for incorporation into DNA origami to be used as carriers. c) Concentration-dependent signals for different concentration of CRP from 500 pg/ml to 10 µg/ml against aptamer 1. d) SPR response plotted against CRP concentration. The solid line is a fit of the Langmuir isotherm to the data with a KD of ~40 nM.

In addition to nanopipette translocation experiments, a gel electrophoresis experiment (2 hours at 80 V at 4°C) was carried out to confirm CRP binding to the carrier (figure S4). Carriers (9 nM) were incubated for 30 min with increasing concentrations of CRP (9 nM, 18 nM, 27 nM, and 36 nM; lanes 4–7). For the lowest CRP concentration, two bands can be seen, one at the same position as the band resulting from the unoccupied carriers (lanes 2 and 8), and one slightly retarded, suggesting that CRP-occupied carriers run through the gel slower than unoccupied carriers. The carrier with a nonspecific aptamer (lane 3) only produces one band at the same position as the unoccupied carriers, confirming that no CRP binding takes places without a CRP-specific DNA aptamer.

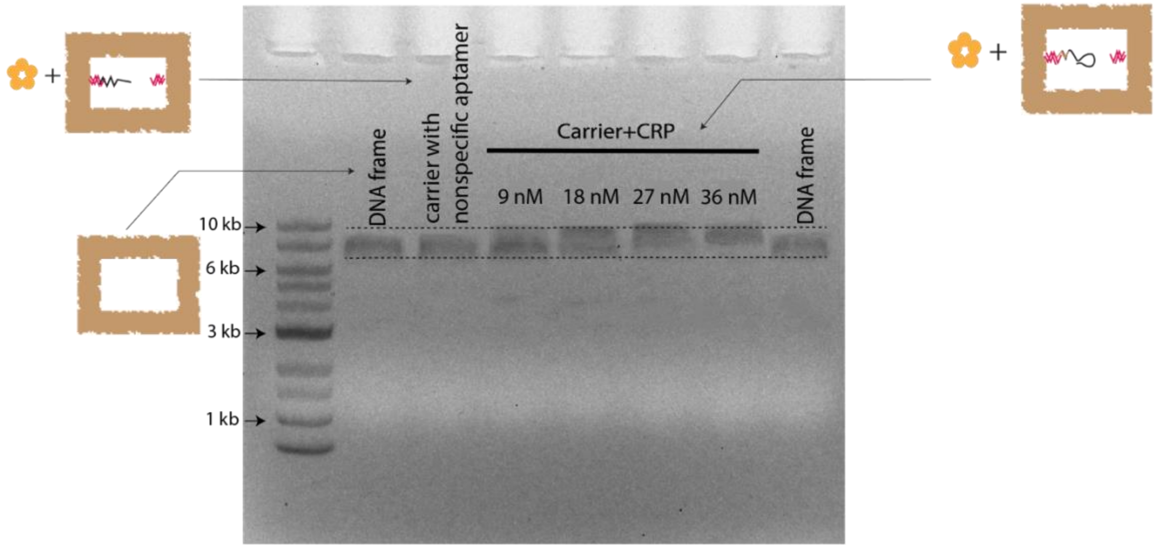

Supplementary Figure 4: Gel retardation assay. Lane 1: 10 kb ladder; lanes 2 and 8: unoccupied carriers; lane 3: carrier with non-CRP-specific aptamer; lanes 4–7: CPR-specific carriers incubated with different concentrations of CRP.

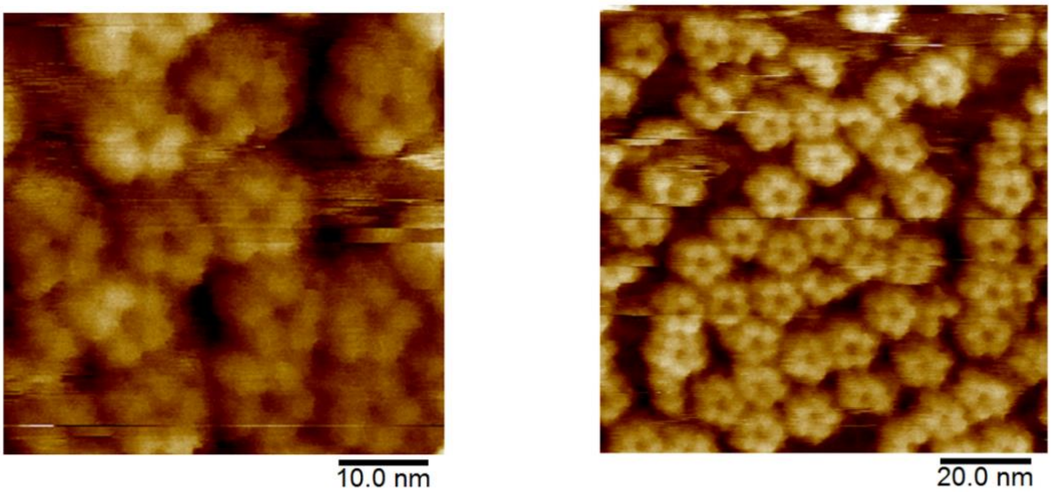

Supplementary Figure 5: AFM micrographs of CRP protein.

Characterisation of carriers

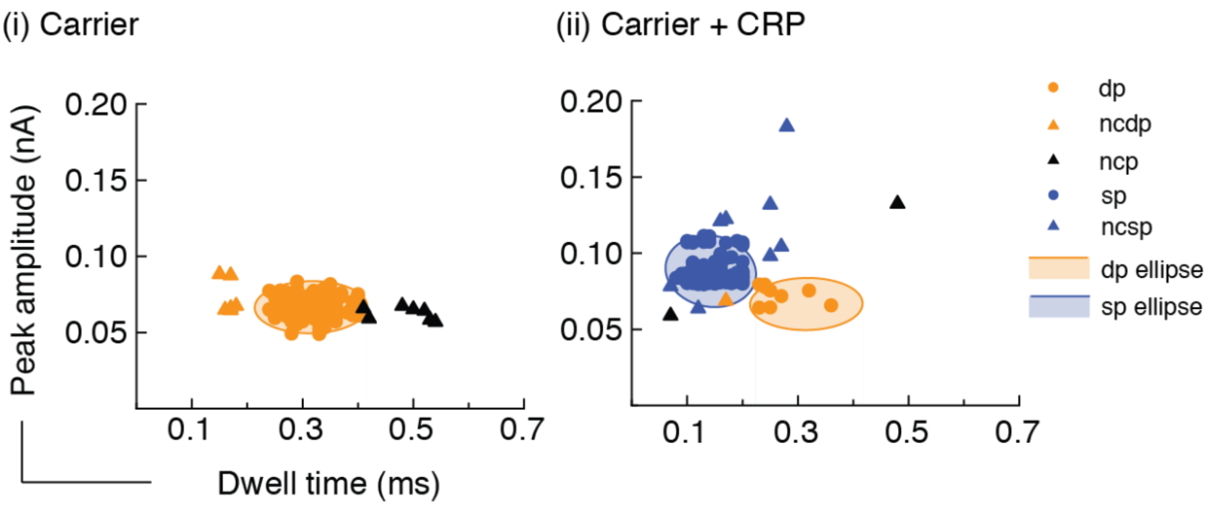

Supplementary Figure 6a: Scatter plot of peaks for (i) unoccupied carriers and (ii) carriers incubated with a 10x excess of CRP (90 nM). Ion current events which are double peaks are plotted orange, and the ones that fall inside the 95% confidence ellipse are plotted as circles, the others as triangles. Only events that fall within the 95% confidence ellipse are considered double peaks. The same analysis was carried out for single peaks (plotted in blue). Ion current events which resembled neither a double nor a single peak are shown as black triangles and are excluded from the analysis. . Legend abbreviations, dp (orange circles) – double peaks, sp (blue circles) – single peaks, ncp (black triangles) – non-classified peaks, ncsp (blue triangles) – non-classified single peaks, ncdp (orange triangles) – non-classified double peaks, dp ellipse (orange ellipse) – 95% confidence ellipse for dp events, sp ellipse (blue ellipse) – 95% confidence ellipse for sp events.

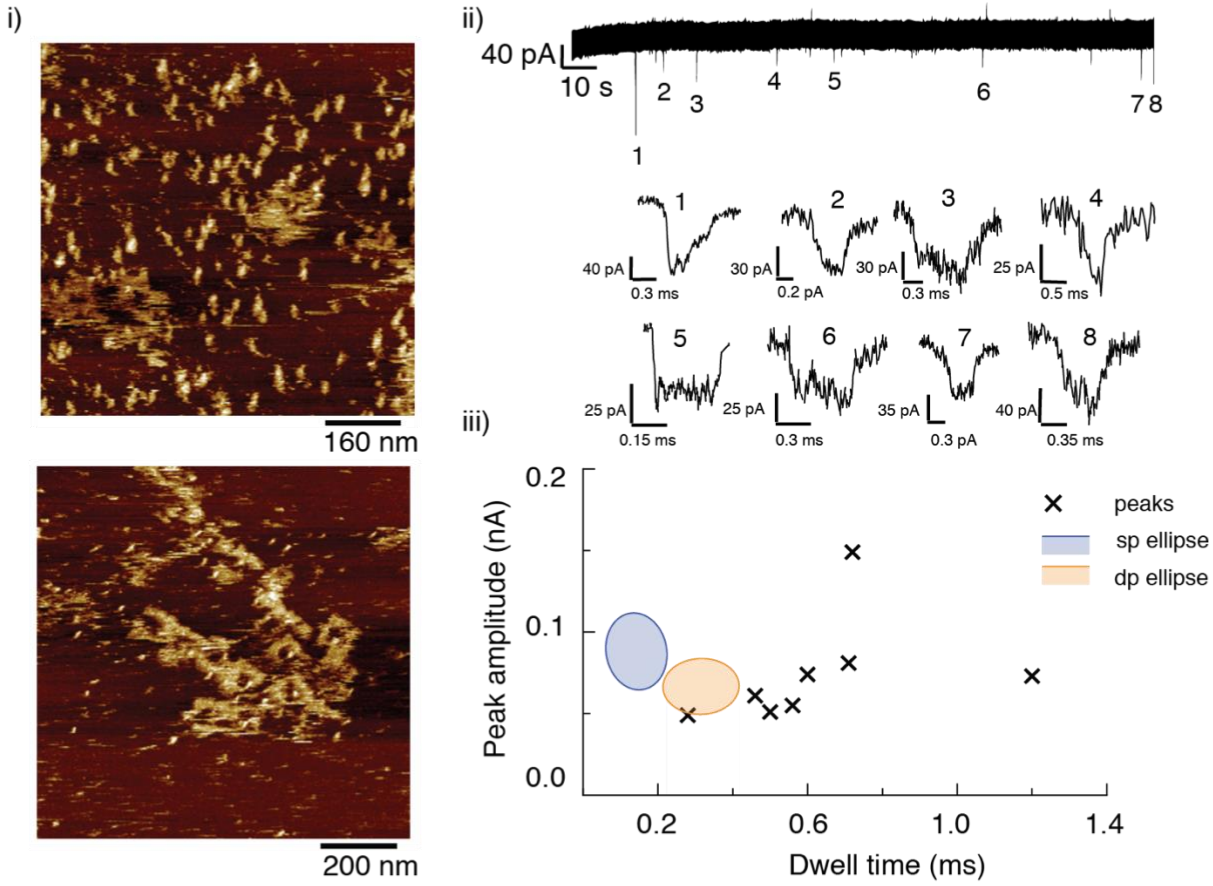

Supplementary Figure 6b: Characteristics of broken carriers. The DNA nanostructures were degraded by incubation in 10 mM $CaCl_2$ at room temperature for 30 minutes. The presence of Ca ions affects the stability of the DNA origami structure by replacing the Mg ions holding the origami together. (i) AFM micrographs showing degraded carriers. (ii) Ion current trace for the degraded samples recorded for about 2 minutes resulted in very few events. A range of different peak shapes were observed. (iii) Scatter plot of peak amplitude versus dwell time, with the 95% confidence ellipses overlaid. All observed peaks (black cross) fall outside the overlaid confidence ellipses. Legend abbreviations, black cross – observed peaks (events), dp ellipse (orange ellipse) – 95% confidence ellipse for dp events, sp ellipse (blue ellipse) – 95% confidence ellipse for sp events.

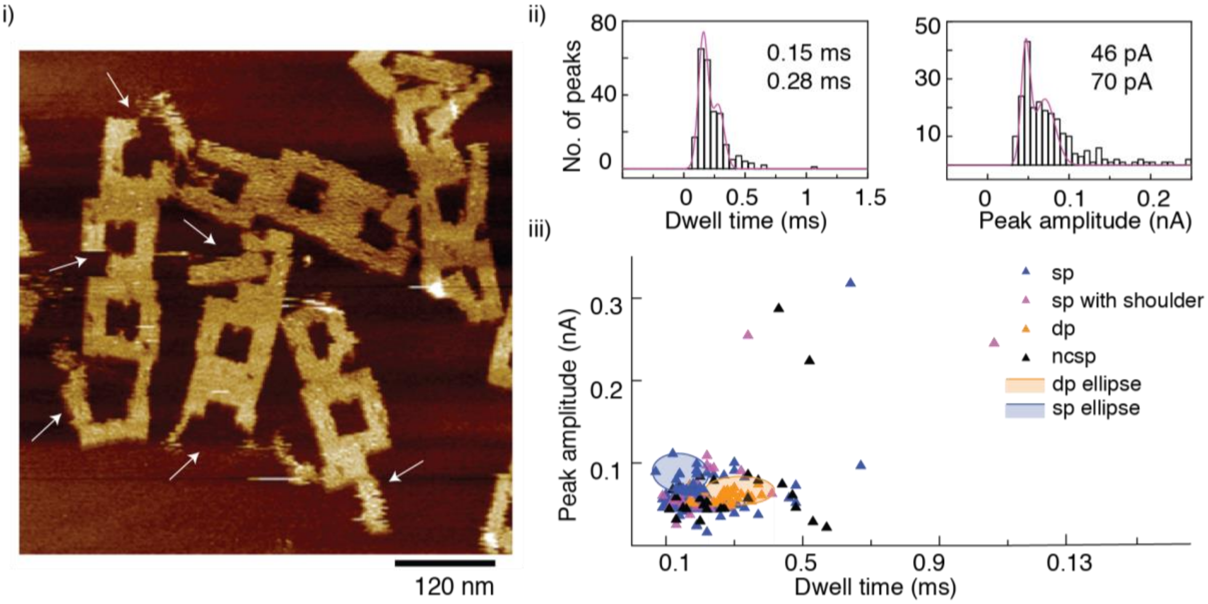

Supplementary Figure 6c: The DNA nanostructures were incubated in translocation buffer at room temperature for 4 hours to assess the impact of the 2 mM Ca^{2+} on the stability of the DNA origami structure. (i) AFM micrograph showing a mixture of intact and degraded carriers (indicated by white arrows). (ii) Peak amplitude and dwell time histograms of the translocation ion current peaks. A range of different peak shapes were observed, including single(blue triangles – sp) and double peaks(orange triangles – dp) and single peaks with shoulders(pink triangles – sp with shoulder). (iii). Scatter plot of peak amplitude versus dwell time, with the 95% confidence ellipses overlaid. Only around 10% of peaks which resemble single peaks also fall into the single peak 95% confidence ellipse and hence would lead to false positives. Legend abbreviations, dp (orange triangles) – double peaks, sp (blue triangles) – single peaks, sp with shoulder (pink triangles) – single peaks with shoulder, ncsp (black triangles) – non-classified single peaks, dp ellipse (orange ellipse) – 95% confidence ellipse for dp events, sp ellipse (blue ellipse) – 95% confidence ellipse for sp events.

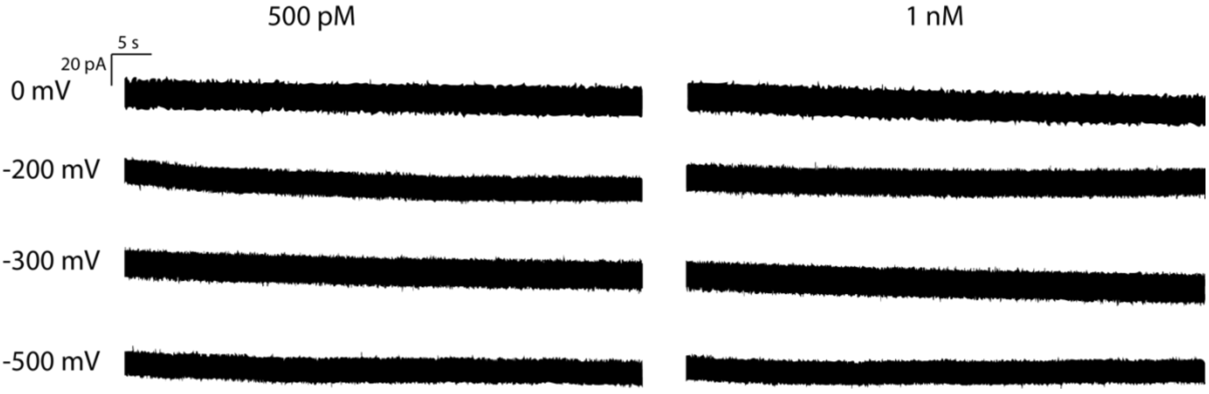

Figure 7a: Ion current traces (~ 1 min) obtained upon translocation of lambda DNA through ~100 nm pore sized nanopipettes for two different concentrations (500 pM and 1 nM) and different voltages. No ion current peaks were observed.

Translocation analysis for different CRP concentrations

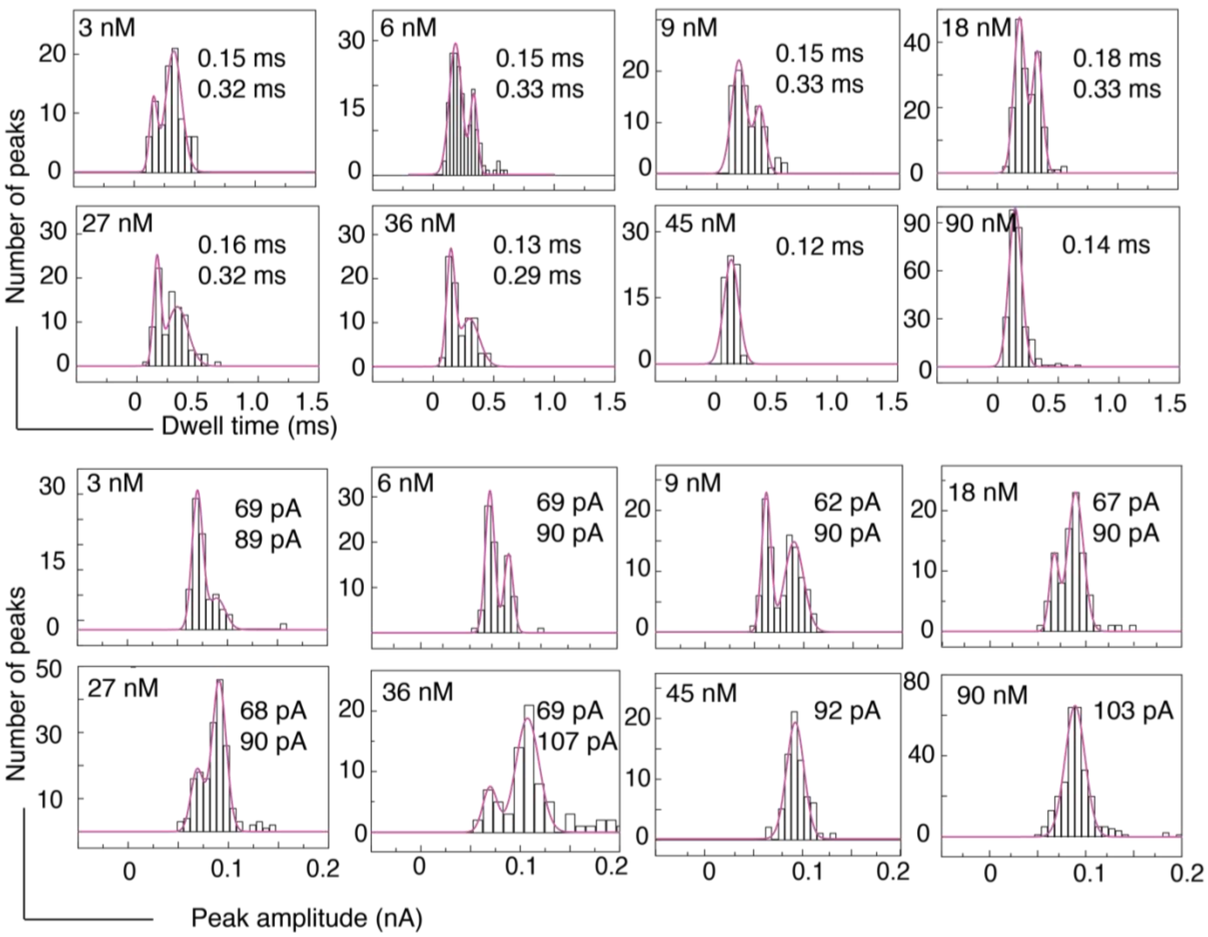

Supplementary Figure 7b: Histograms of dwell time and peak amplitude for the carriers made with aptamer 1 incubated with different concentrations of CRP (3 to 90 nM).

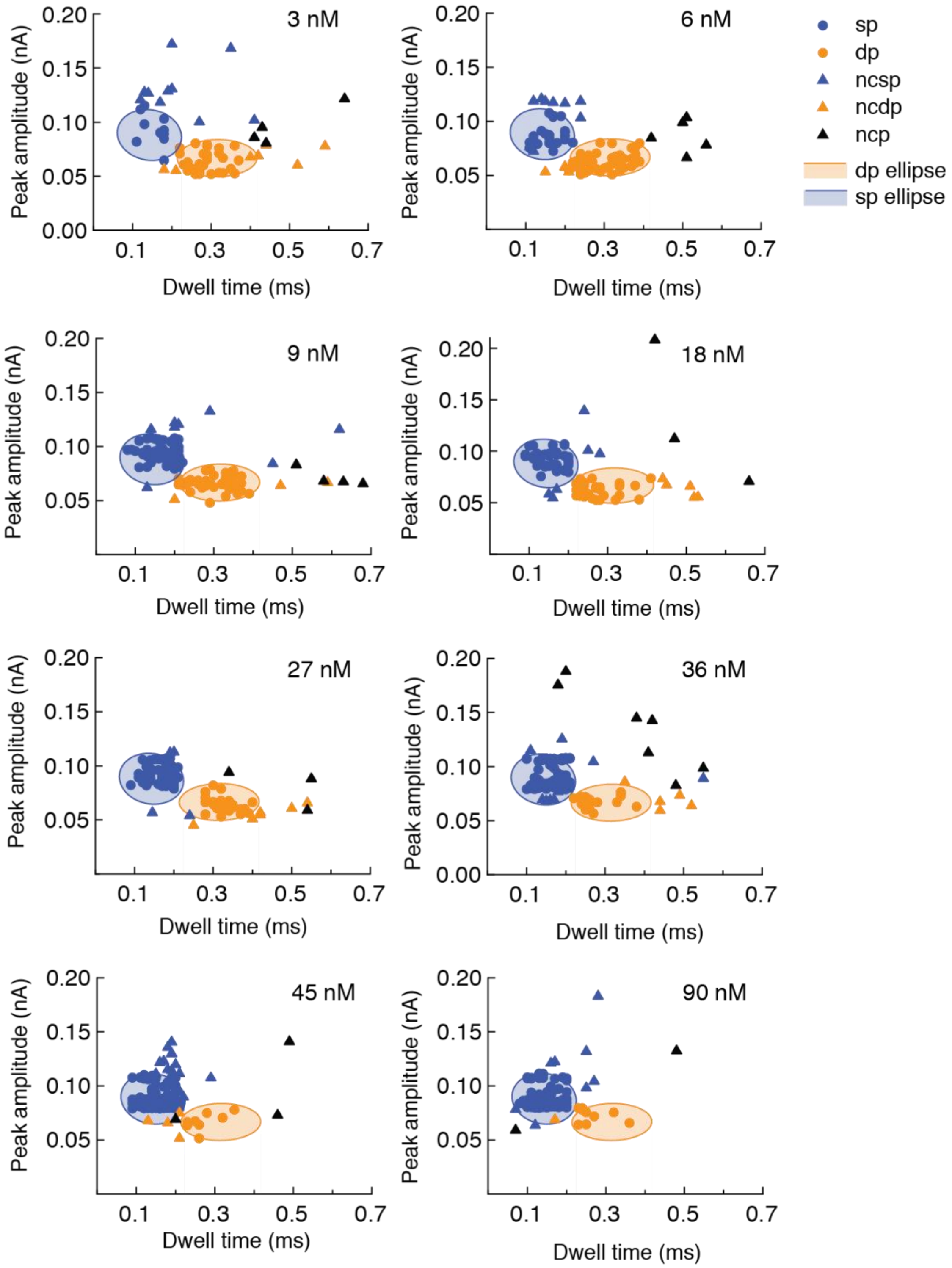

Supplementary Figure 7c: Scatter plots of ion current events – peak amplitude versus dwell time – for carriers made with aptamer 1 incubated with different CRP concentration (3 to 90 nM). The individual observed ion current peaks are classified as described in the main text and colour-coded (double peaks orange and single peaks blue, triangles represent unclassified events which are not considered for analysis). The orange and blue ellipses represent the 95% confidence ellipses for double and single peaks and are taken from the analysis described in figure S6a. The panels for 9 nM and 90 nM are the same as presented in figure 3b. Legend abbreviations, dp (orange circles) – double peaks, sp (blue circles) – single peaks, ncp (black triangles) – non-classified peaks, ncsp (blue triangles) – non-classified single peaks, ncdp (orange triangles) – non-classified double peaks, dp ellipse (orange ellipse) – 95% confidence ellipse for dp events, sp ellipse (blue ellipse) – 95% confidence ellipse for sp events.

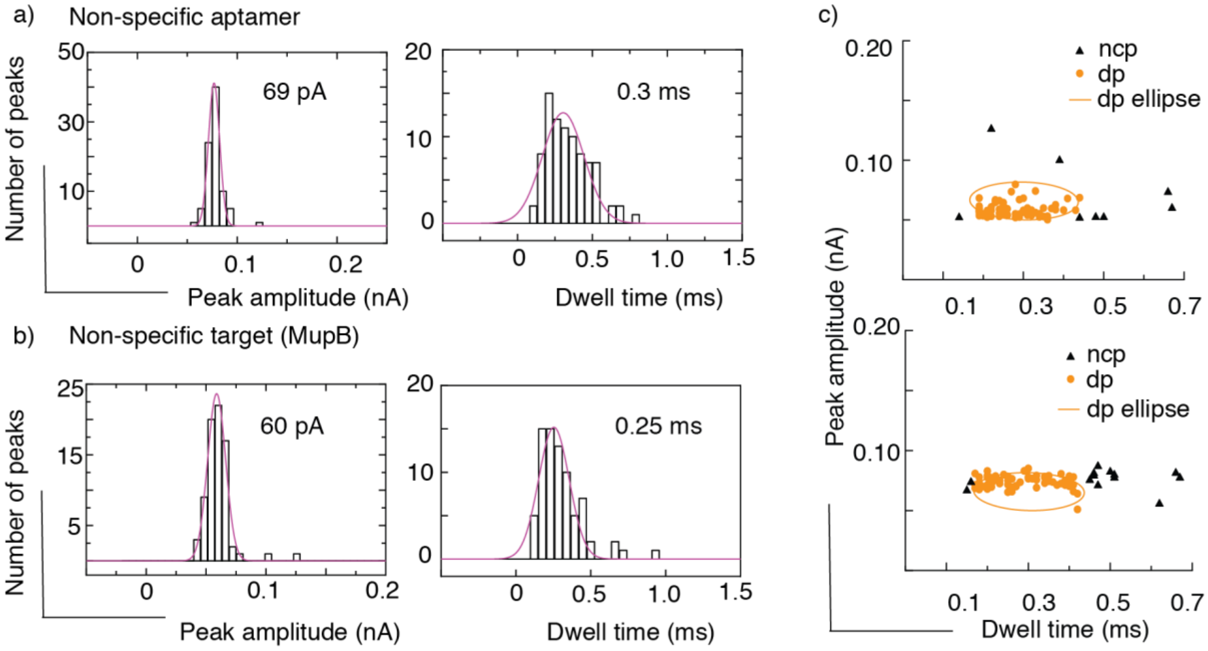

Supplementary Figure 8: a) Ion current peak analysis for carrier made with non-specific aptamer challenged with 90 nM CRP. Experimental conditions are the same as used for specific sensing at the highest CRP concentration, i.e. 9 nM carrier and 90 nM CRP. A peak amplitude and dwell time of 69±5 pA and 0.3±0.13 ms, respectively, were found, which is in line with those for an empty carrier. The scatter plot analysis which shows the classification of the ion current events as described in the main text shows that no occupied carriers were detected, demonstrating the high specificity of the sensing approach. b) Ion current peak analysis for CRP-specific carriers challenged with 90 nM of a different protein (MupB). Experimental conditions are the same as used for specific sensing at the highest CRP concentration, i.e. 9 nM carrier and 90 nM MupB. A peak amplitude and dwell time of 60±7 pA and 0.25±0.1 ms, respectively, were found, which is again similar to those for an empty carrier. c) The scatter plot analysis which shows the classification of the ion current events as described in the main text shows that no occupied carriers were detected, demonstrating the high specificity of the sensing approach. Legend abbreviations, dp (orange circles) – double peaks, ncp (black triangles) – non-classified peaks, dp ellipse (orange ellipse) – 95% confidence ellipse for dp events.

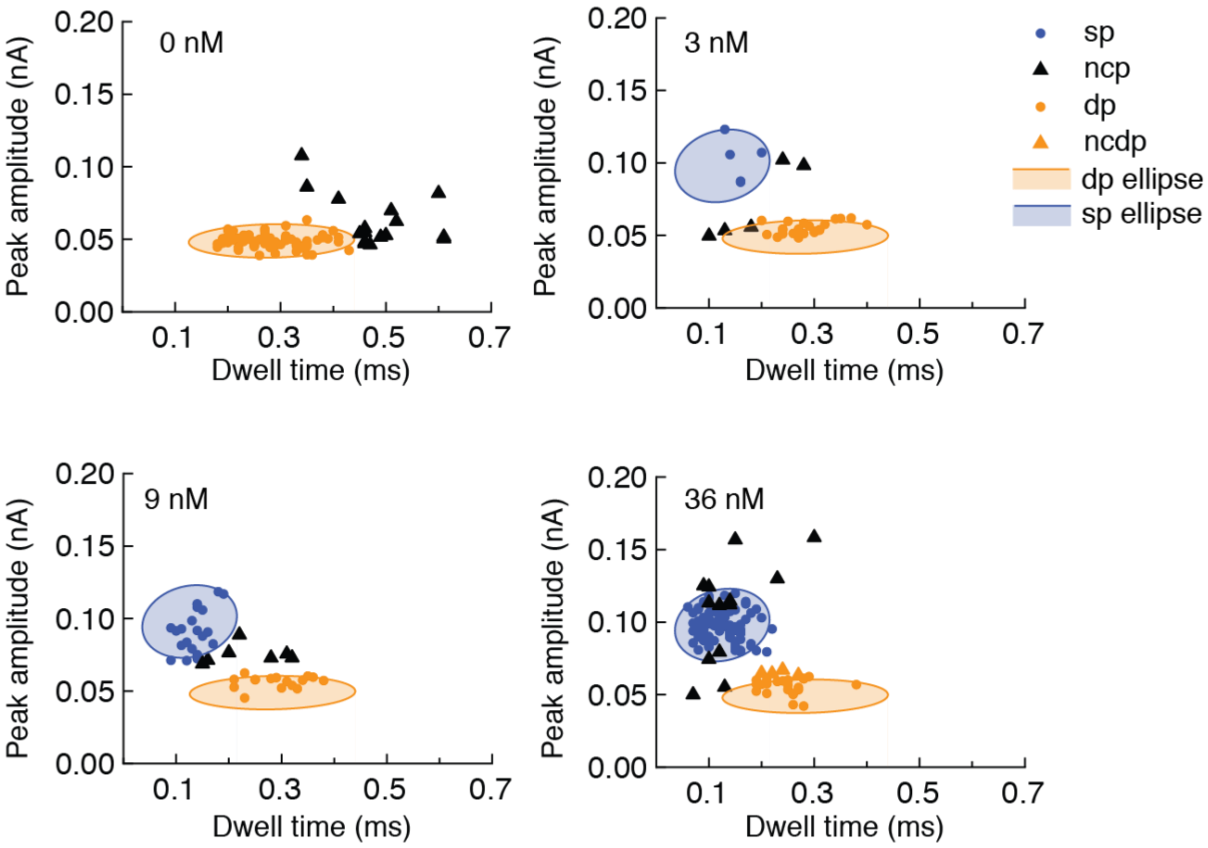

Supplementary Figure 9: Scatter plot of peak amplitude versus dwell time for carriers (9 nM) subjected to four different concentrations of CRP (0 nM, 3 nM, 9 nM and 36 nM) in plasma. For 0 nM, no single peaks were observed, and the cluster of double peaks was used to define the 95% confidence interval for the unoccupied carrier. Similarly, the 95% confidence ellipse for the single peaks was generated from the data of the highest CRP concentration. Legend abbreviations, dp (orange circles) – double peaks, sp (blue circles) – single peaks, ncp (black triangles) – non-classified peaks, ncdp (orange triangles) – non-classified double peaks, dp ellipse (orange ellipse) – 95% confidence ellipse for dp events, sp ellipse (blue ellipse) – 95% confidence ellipse for sp events.

Nanopipette characterisation

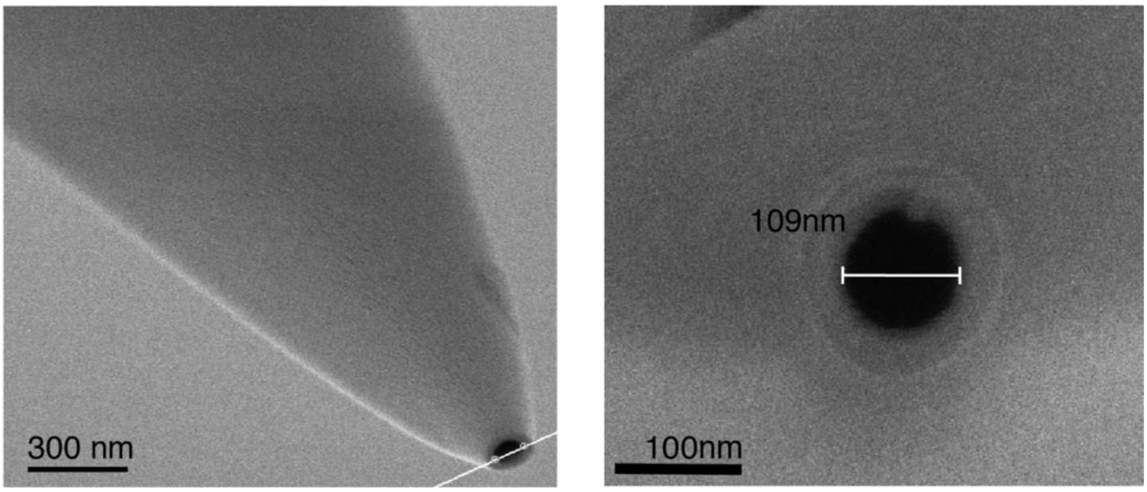

Supplementary Figure 10a: SEM image of a representative nanopipette tip with a pore diameter of approximately 100 nm.

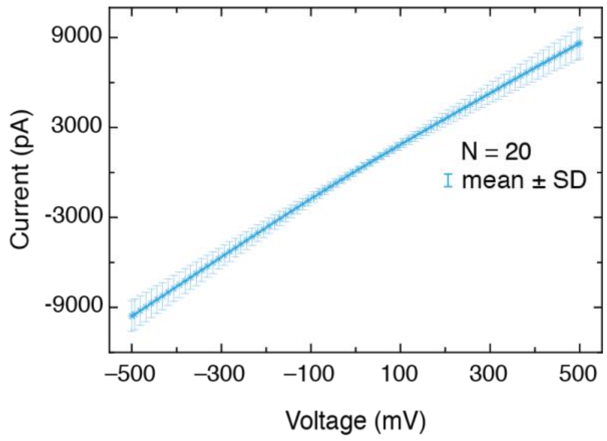

Supplementary Figure 10b: Representative ion current – voltage curves for 20 nanopipettes used in this study. The ion current was recorded in 0.1M KCl. The error bars denote the center mean ± standard deviation.

Single-stranded DNA scaffold design and routing map for concentric squares origamis

For this study, a modified phagemid (plasmid with m13 region) was used to create the three scaffolds of varying lengths to create the DNA origamis with similar outer but different cavity dimensions. The scaffolds required for the three DNA origamis (concentric squares (Con) A, B and C) were stitched together from two separate fragments derived from lambda phage DNA along with pBluescript II SK(+) phagemid (PBS). The PBS(+) are plasmids containing the phage origin of replication (f1 ori) and an antibiotic resistance gene which allows for sense strand rescue by a helper phage. The 9 kb custom scaffold required for the DNA origami ConA was created by inserting a 6.1kb sequence, derived as two separate fragments from lambda phage, into the 2.9 kb phagemid. These two fragments (approximately 1.8 kb and 4.2 kb) were selected devoid of protein coding regions to eliminate any interference in the phage growth and downstream process. The map for scaffold assembly is provided in Figure S11b and S11c.

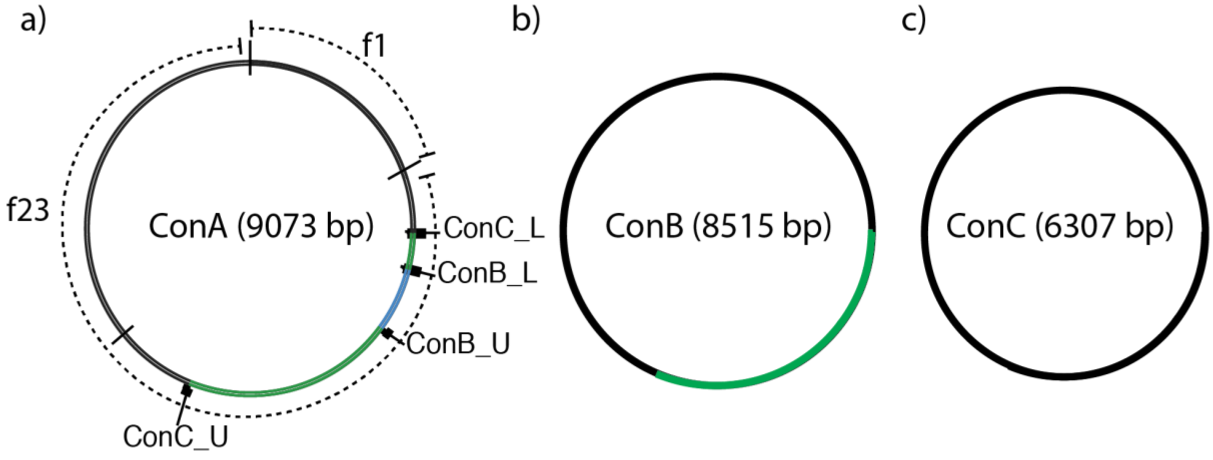

Supplementary Figure 11a: Schematic of concentric square scaffold design. a) Scaffold for ConA DNA origami tile indicated with the upper (ConB_U, ConC_U) and lower (ConB_L, ConC_L) primers for deriving b) ConB and c) ConC scaffolds respectively.

The three fragments that make up the scaffold – referred to as f1, f2 and f3 – were PCR amplified from lambda phage DNA and the PBS plasmid, respectively, using appropriate primers. The primers used in the amplification procedure were designed to incorporate the restriction sites EcoRI and NspI flanking f2, and NcoI and NspI flanking f3. The two fragments were then restriction digested with the respective enzymes to form compatible overhangs, and then ligated to yield one long fragment (f23). Fragment f1 was then combined with fragment f23 using HiFi DNA assembly cloning (NEB,UK), resulting in the double stranded circular f123 DNA.

Subsequently the circular dsDNA (f123) was chemically transformed in competent *E.Coli* cells to produce the custom scaffold. The transformed cells with the f123 plasmid were selected using their antibiotic resistance gene and recovered with a miniprep to be co-transformed with helper plasmids (m13cp cells) to produce ssDNA f123. The final ssDNA f123 product constituted 9073 bp and was used as the scaffold for the largest DNA construct ConA.

The scaffolds for the two smaller DNA constructs, ConB and ConC, were produced by PCR amplification of the double stranded f123 as template. As before the primers were designed to incorporate the restriction site KasI for the product ConB and BmtI for the product ConC. Subsequent restriction and ligation followed by similar co-transformation protocol with helper plasmids as above resulted in 8515 bp and 6307 bp ssDNA scaffolds for Con B and C, respectively. In the first instance, the scaffolds were produced in 5 ml cultures and subsequently followed by large-scale scaffold harvesting by scaling up to 1-litre cultures. The cultures were harvested by separating the bacteria from the phage and subsequent phenol chloroform DNA extraction.

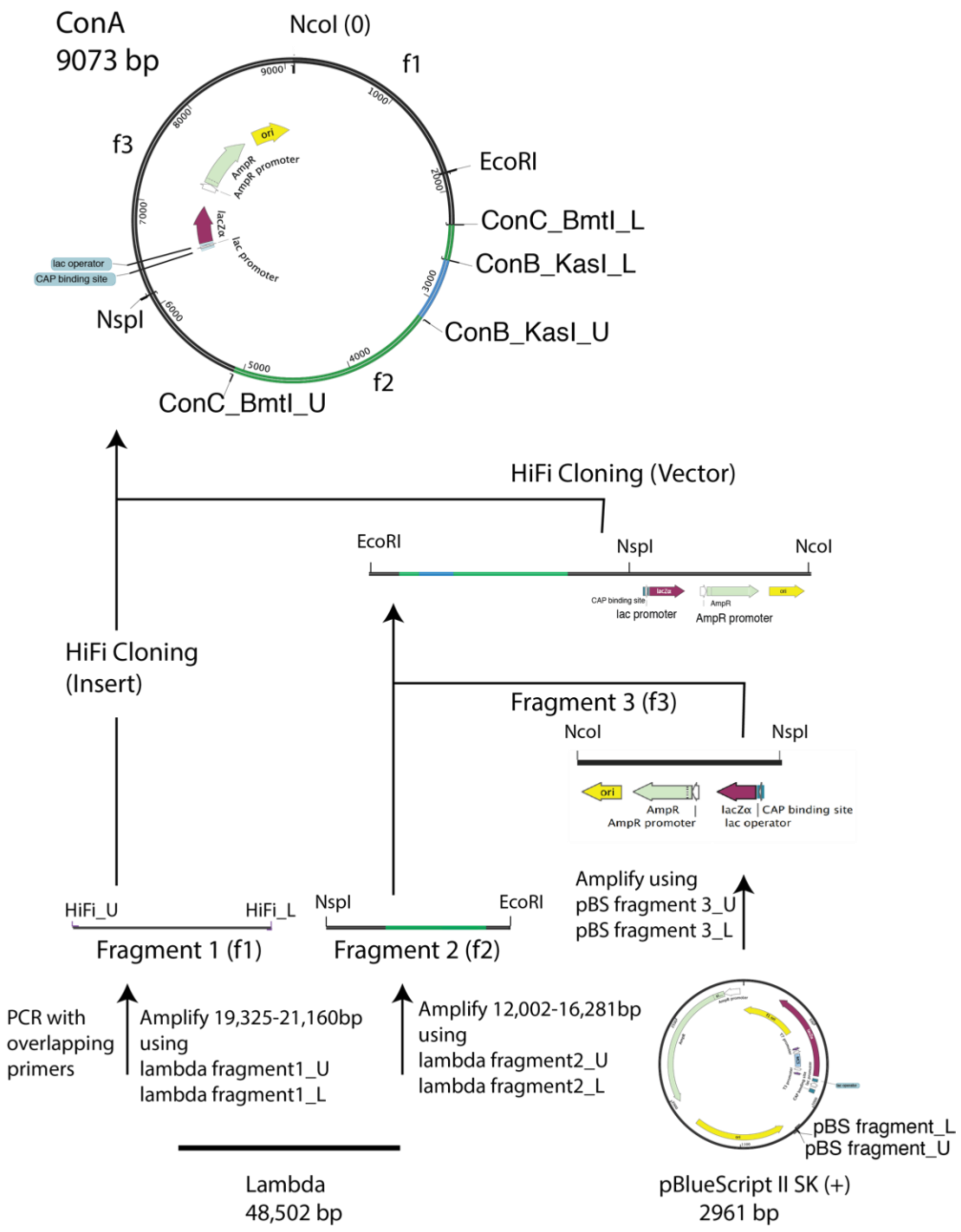

Supplementary Figure 11b: Concentric square scaffold map and construction workflow.

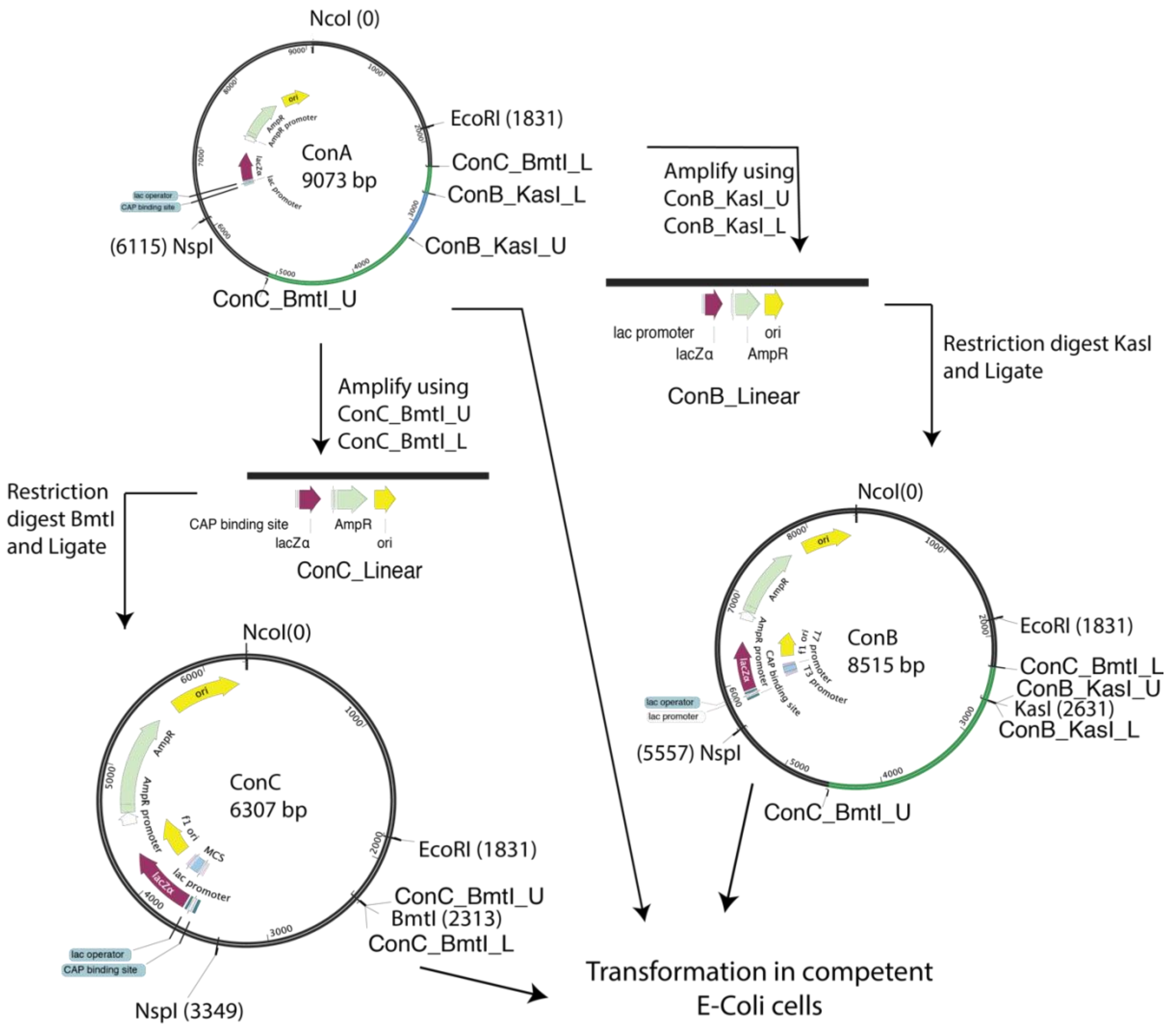

Supplementary Figure 11c: PCR amplification of ConB and ConC scaffold from ConA scaffold.

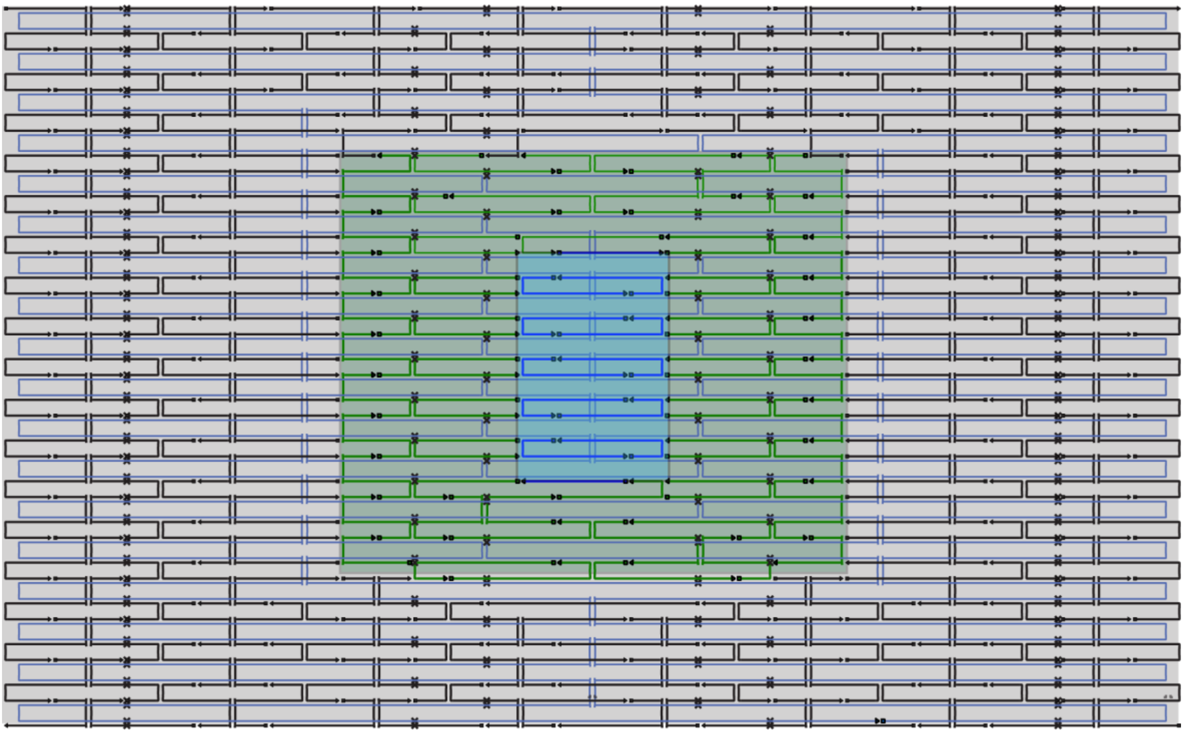

Supplementary figure 11d: CaDNAno scaffold routing of concentric squares (ConA, ConB, and ConC). The full tile comprising the black, green and blue shaded regions represents the ConA DNA origami, whereas excluding the blue shaded region in the centre from the full tile represents ConB, and ConC consists of the black shaded region alone.

Biosensor with CRP-specific aptamer 2

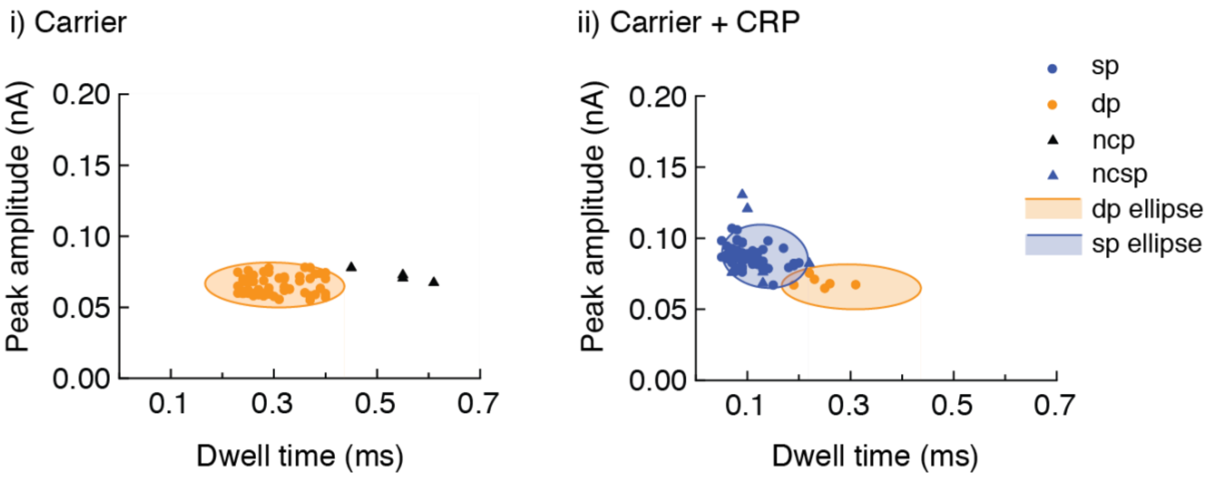

Supplementary figure 12: Scatter plot of peaks for (i) unoccupied carriers and (ii) carriers (6 nM) incubated with a 17x excess of CRP (100 nM). Ion current events which are double peaks are plotted orange, and the ones that fall inside the 95% confidence ellipse are plotted as circles, the others as triangles. Only events that fall within the 95% confidence ellipse are considered double peaks. The same analyses were carried out for single peaks (plotted in blue). Ion current events which resembled neither a double nor a single peak are shown as black triangles and are excluded from the analysis. Legend abbreviations, dp (orange circles) – double peaks, sp (blue circles) – single peaks, ncp (black triangles) – non-classified peaks, ncsp (blue triangles) – non-classified single peaks, dp ellipse (orange ellipse) – 95% confidence ellipse for dp events, sp ellipse (blue ellipse) – 95% confidence ellipse for sp events.

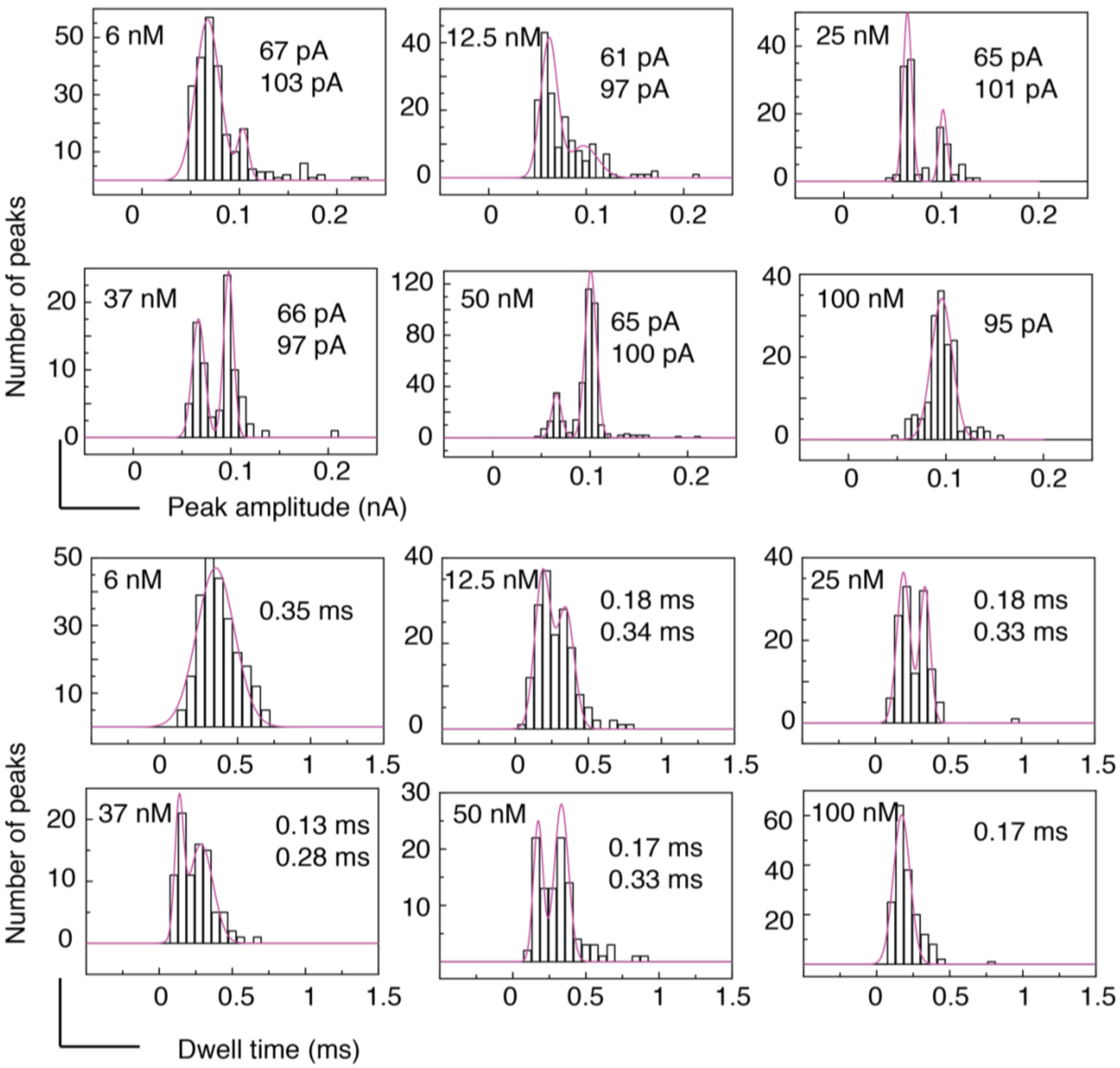

Supplementary Figure 13a: Individual histograms of dwell time and peak amplitude observed for carriers incubated with varying concentrations of CRP (6 nM to 100 nM).

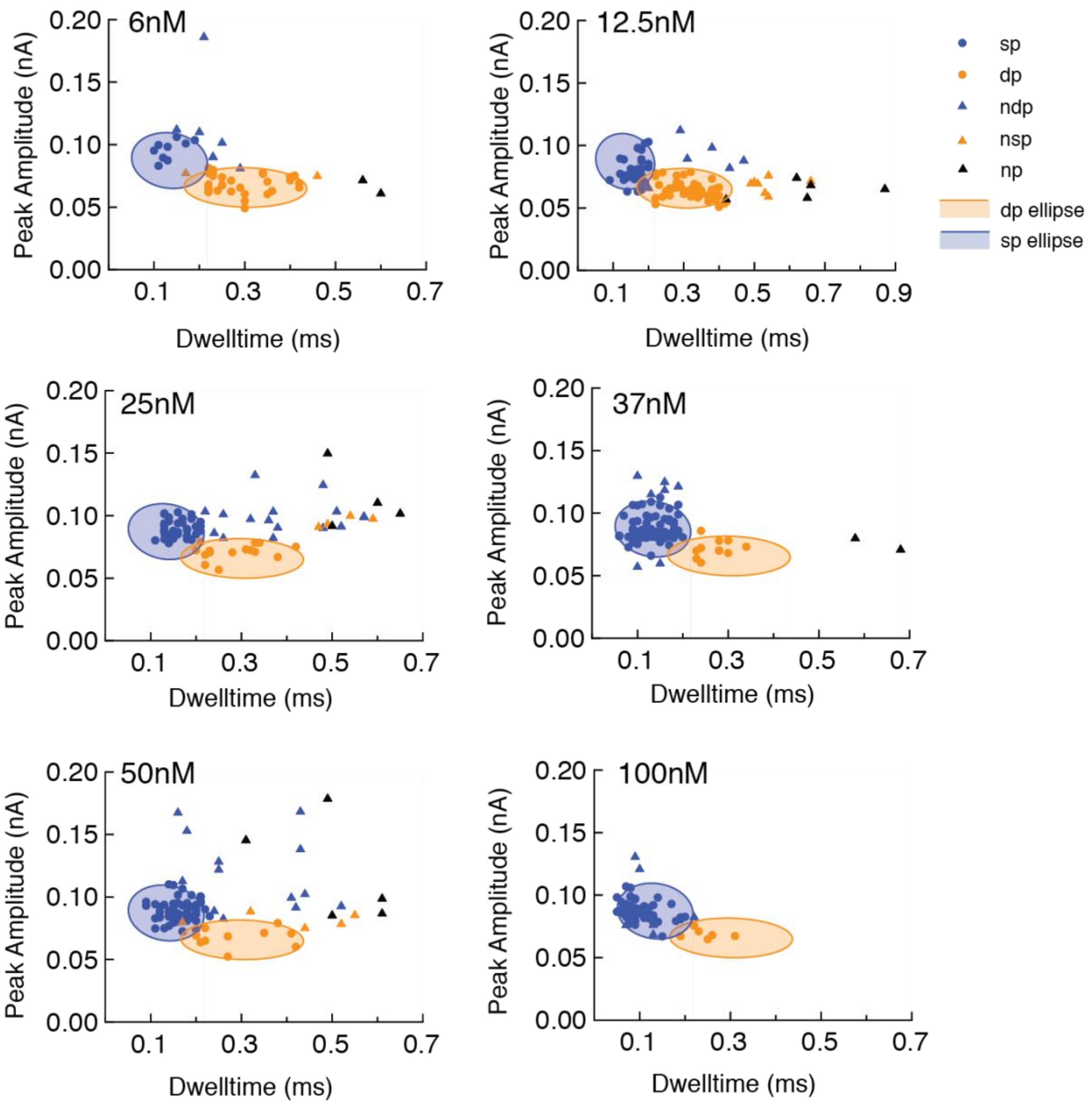

Supplementary Figure 13b: Scatter plot of ion current events plotted as peak amplitude versus dwell time for carriers incubated with different CRP concentration (6 to 100 nM). The ion peak signatures of double peak and single peaks observed for the samples are colour-coded orange and blue respectively and indicated by a 95% confidence eclipse obtained from figure S12, unclassified events are represented as triangles. Legend abbreviations, dp (orange circles) – double peaks, sp (blue circles) – single peaks, ncp (black triangles) – non-classified peaks, ncsp (blue triangles) – non-classified single peaks, ncdp (orange triangles) – non-classified double peaks, dp ellipse (orange ellipse) – 95% confidence ellipse for dp events, sp ellipse (blue ellipse) – 95% confidence ellipse for sp events.

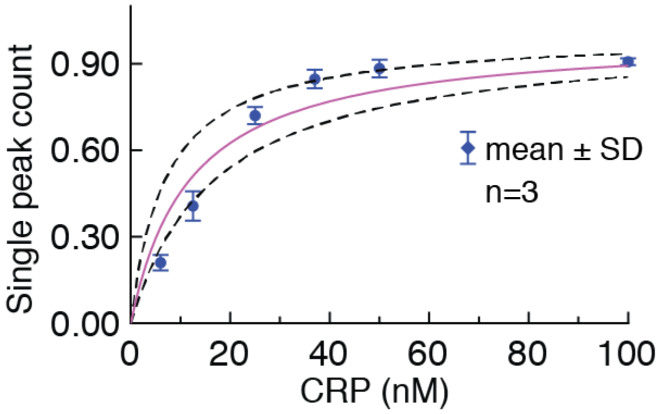

Supplementary figure 13c: Normalised single peak count, i.e. ratio of single peaks vs total classified peaks against CRP concentration (table S5). The data were fitted with Langmuir isotherm (solid line) and revealed a K_d of 12±5 nM. The dashed lines represent the confidence boundaries of the fit. The error bars denote standard deviation of ion current translocation experiments conducted on different days using three different nanopipettes.

Schematic of DNA origami carrier design

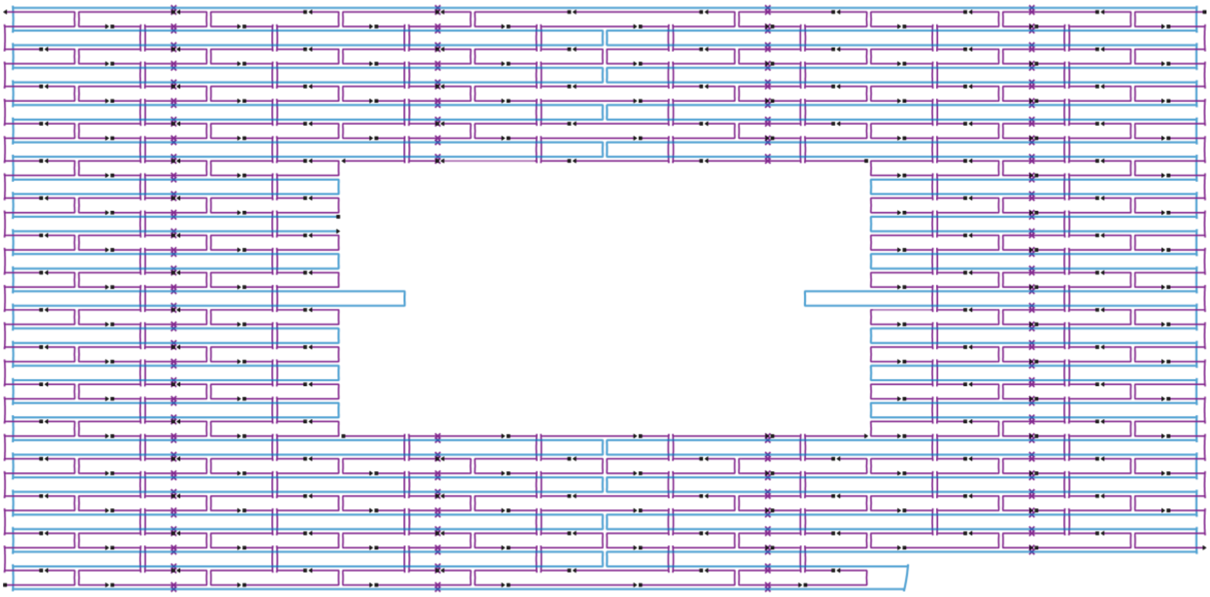

Supplementary figure 14: CaDNAno scaffold routing of the DNA origami frame used as the carrier. The scaffold strand is coloured blue whereas the numerous short staple stranded are presented in pink.

Supplementary Tables

Supplementary Table 1: Normalised single peak counts (single peaks/(single peaks+double peaks)) for 2-minute ion current traces for 3 different nanopipettes. The carrier concentration was 9 nM. This table is compiled from the data provided in table S2.

CRP (nM)	Pipette 1	Pipette 2	Pipette 3	average	std
3	0.25	0.2	0.19	0.21	0.03
6	0.29	0.34	0.26	0.29	0.04
9	0.47	0.42	0.39	0.42	0.04
18	0.57	0.57	0.48	0.54	0.05
27	0.71	0.67	0.67	0.68	0.02
36	0.83	0.77	0.89	0.83	0.06
45	0.94	0.81	0.89	0.88	0.07
90	0.92	0.89	0.91	0.90	0.02

Supplementary Table 2: Number of single peaks, double peaks and unclassified events observed for carriers incubated with different concentrations of CRP for 3 different nanopipettes measured on different days. sp – single peaks, dp – double peaks, ncp – non-classified peaks.

CRP (nM)	Pipette 1				Pipette 2				Pipette 3			
	sp	dp	ncp	total	sp	dp	ncp	total	sp	dp	ncp	total
3	10	29	21	60	9	36	11	56	9	38	13	60
6	19	46	18	83	18	34	30	82	12	34	16	62
9	46	52	16	114	19	26	12	57	31	49	27	107
18	41	31	14	86	19	14	17	50	32	35	17	84
27	58	24	13	95	36	18	15	69	39	19	12	70
36	84	17	17	118	67	20	36	123	58	7	24	89
45	132	8	22	162	59	14	9	82	72	9	16	97
90	105	9	11	125	48	6	13	67	64	6	11	81

Supplementary Table 3: Normalised single peak counts (single peaks/ (single peaks+double peaks)) for 3 different ion current traces. The carrier concentration was 9 nM. This table is compiled from the data provided in table S4.

CRP (nM)	Trace 1	Trace 2	Trace 3	average	std
3	0.14	0.13	0.18	0.15	0.02
9	0.61	0.64	0.55	0.6	0.04
36	0.95	0.84	0.77	0.85	0.09

Supplementary Table 4: Number of single peaks, double peaks and unclassified events observed for carriers incubated with different concentrations of CRP for 3 in 5% human plasma for three different ~2 minute ion current traces. sp – single peaks, dp – double peaks, ncp – non-classified peaks.

CRP (nM)	trace1				trace2				trace3			
	sp	sp	total	ncp	sp	dp	total	ncp	sp	dp	total	ncp
3	5	22	32	5	4	27	41	5	4	23	30	3
9	20	16	43	7	46	25	87	16	24	15	52	13
36	90	26	128	12	117	21	142	4	61	3	69	5

Supplementary table 5: Normalised single peak counts (single peaks/(single peaks+double peaks)) for 2-minute ion current traces for 3 different nanopipettes. The carrier concentration was 6 nM. This table is compiled from the data provided in table S6.

CRP (nM)	Pipette 1	Pipette 2	Pipette 3	average	std
6	0.28	0.38	0.28	0.31	0.06
12.5	0.44	0.36	0.45	0.42	0.05
25	0.62	0.60	0.67	0.63	0.03
37	0.77	0.75	0.86	0.79	0.06
50	0.88	0.82	0.94	0.88	0.06
100	0.95	0.96	0.93	0.95	0.01

Supplementary Table 6: Number of single peaks, double peaks and unclassified events observed for carriers incubated with different concentrations of CRP for 3 different nanopipettes measured on different days. sp – single peaks, dp – double peaks, ncp – non-classified peaks.

CRP (nM)	pipette 1				pipette 2				pipette 3			
	sp	dp	ncp	total	sp	dp	ncp	total	sp	dp	ncp	total
6	8	31	12	51	12	37	9	58	8	33	13	54
12.5	35	48	19	102	17	31	17	65	24	30	11	65
25	40	15	25	80	30	13	28	71	35	12	11	58
37	78	11	10	99	38	9	22	69	55	9	12	76
50	58	10	23	91	174	22	39	235	77	8	22	107
100	58	6	6	70	53	4	12	57	111	11	22	144
