# Supplementary material for: Rational design of DNA nanostructures for single molecule biosensing
Source: Nat Commun. 2020 Sep 1;11:4384. doi: 10.1038/s41467-020-18132-1 (PMC7463249; doi:10.1038/s41467-020-18132-1)
Supplement: Supplementary file 2 — Reporting Summary [file 41467_2020_18132_MOESM2_ESM.pdf]

nature research

Corresponding author(s): Paolo Actis

Last updated by author(s): Jul 27, 2020

Reporting Summary

Nature Research wishes to improve the reproducibility of the work that we publish. This form provides structure for consistency and transparency in reporting. For further information on Nature Research policies, see our Editorial Policies and the Editorial Policy Checklist.

Statistics

For all statistical analyses, confirm that the following items are present in the figure legend, table legend, main text, or Methods section.

n/a	Confirmed	
[ ]	[x]	The exact sample size (*n*) for each experimental group/condition, given as a discrete number and unit of measurement
[ ]	[x]	A statement on whether measurements were taken from distinct samples or whether the same sample was measured repeatedly
[x]	[ ]	The statistical test(s) used AND whether they are one- or two-sided<br>*Only common tests should be described solely by name; describe more complex techniques in the Methods section.*
[x]	[ ]	A description of all covariates tested
[ ]	[x]	A description of any assumptions or corrections, such as tests of normality and adjustment for multiple comparisons
[ ]	[x]	A full description of the statistical parameters including central tendency (e.g. means) or other basic estimates (e.g. regression coefficient) AND variation (e.g. standard deviation) or associated estimates of uncertainty (e.g. confidence intervals)
[x]	[ ]	For null hypothesis testing, the test statistic (e.g. *F*, *t*, *r*) with confidence intervals, effect sizes, degrees of freedom and *P* value noted<br>*Give P values as exact values whenever suitable.*
[x]	[ ]	For Bayesian analysis, information on the choice of priors and Markov chain Monte Carlo settings
[x]	[ ]	For hierarchical and complex designs, identification of the appropriate level for tests and full reporting of outcomes
[x]	[ ]	Estimates of effect sizes (e.g. Cohen's *d*, Pearson's *r*), indicating how they were calculated

Our web collection on statistics for biologists contains articles on many of the points above.

Software and code

Policy information about availability of computer code

Data collection	Pclamp 10.6 for ion current measurements, nanoscope 9.1 for AFM micrographs, caDNAno 2.3 for designing DNA nanostructures.
Data analysis	Ion current data analysis was performed with the Matlab (software version 9.5) script provided by Prof Joshua Edel (Imperial College, London), which has been used in 10+ previous publications. proFit 7 software was also made use of for the ion current data analysis. AFM micrographs were analysed via the nanoscope analysis 1.9 software.

For manuscripts utilizing custom algorithms or software that are central to the research but not yet described in published literature, software must be made available to editors and reviewers. We strongly encourage code deposition in a community repository (e.g. GitHub). See the Nature Research guidelines for submitting code & software for further information.

Data

Policy information about availability of data

All manuscripts must include a data availability statement. This statement should provide the following information, where applicable:

- Accession codes, unique identifiers, or web links for publicly available datasets
- A list of figures that have associated raw data
- A description of any restrictions on data availability

Data supporting this work can be accessed via the University of Leeds repository, https://doi.org/10.5518/858

Field-specific reporting

Please select the one below that is the best fit for your research. If you are not sure, read the appropriate sections before making your selection.

- [x] Life sciences
- [ ] Behavioural & social sciences
- [ ] Ecological, evolutionary & environmental sciences

For a reference copy of the document with all sections, see nature.com/documents/nr-reporting-summary-flat.pdf

Life sciences study design

All studies must disclose on these points even when the disclosure is negative.

Sample size

No sample size calculation was performed. The sample sizes used in this study are consistent with previously published nanopore translocation works. For example, depending on the type of sample and translocation conditions; the sample number N (number of translocation events) ranged from 30 to 235. Where as, for reproducibility studies using different nanopipettes and ion current traces, the sample number N was 3.

Few recent references :

1. Cai, S., Sze, J. Y. Y., Ivanov, A. P. & Edel, J. B. Small molecule electro-optical binding assay using nanopores. Nat. Commun. 10, 1797 (2019).
2. Chen, K., Zhu, J., Bošković, F. & Keyser, U. F. Nanopore-Based DNA Hard Drives for Rewritable and Secure Data Storage. Nano Lett. (2020).
3. Bell, N. A. W., Chen, K., Ghosal, S., Ricci, M. & Keyser, U. F. Asymmetric dynamics of DNA entering and exiting a strongly confining nanopore. Nat. Commun. 8, 1–8 (2017).
4. Lin, X., Ivanov, A. P. & Edel, J. B. Selective single molecule nanopore sensing of proteins using DNA aptamer-functionalised gold nanoparticles. Chem. Sci. 8, 3905–3912 (2017)
5. Freedman, K. J. et al. Nanopore sensing at ultra-low concentrations using single-molecule dielectrophoretic trapping. Nat. Commun. 7, 10217 (2016).
6. Wang, V., Ermann, N. & Keyser, U. F. Current Enhancement in Solid-State Nanopores Depends on Three-Dimensional DNA Structure. Nano Lett. 19, 5661–5666 (2019).

Data exclusions

Exclusion criteria were pre-established and all excluded data are still shown in the data analysis tables and figures.
In the 3-parameter classification experiments, in order to establish a reliable method of classifying different ion current events, a 95% confidence ellipse was fitted to the ion current events to exclude outliers and to ensure robust classification. Thus the events that fall outside the boundary were taken as non-classified peaks (ncsp-non classified single peaks, ncdp- non classified double peaks, ncp-non classified peaks), these events are represented as triangles in the figures (main text figure 3, Supplementary figures 6a,b,c; 7c; 8; 9; 12 and 13b). The number of such excluded events are also provided in the SI tables. This way of data exclusion is described in detail in the paper's main text.

Replication

The nanopipette translocation experiments were replicated with at least three different nanopipettes on different days. The experiments made use of different batches of carrier DNA nanostructures that were folded on different days. The results from these experiments validated the reproducibility of our experiments. Also the robustness of the study was further successfully validated by using 2 different DNA aptamers specific to the target molecule (CRP).

Randomization

Randomization was not relevant to our study. The experimental procedures employed in our study required a sequential procedure, i.e. nanopipettes were fabricated first, the relevant samples were then loaded into the nanopipettes and ion current recording was performed. This was followed by data analysis.

Blinding

Blinding was not applied to our study as is common for this type of studies. The aim of this study was to establish a quantitative single molecule biosensing platform, which was achieved by individually counting the number of ion current peaks (single and double peaks) observed over various concentrations of target with respect to the carrier concentration.

Reporting for specific materials, systems and methods

We require information from authors about some types of materials, experimental systems and methods used in many studies. Here, indicate whether each material, system or method listed is relevant to your study. If you are not sure if a list item applies to your research, read the appropriate section before selecting a response.

Materials & experimental systems

n/a	Involved in the study
x	Antibodies
x	Eukaryotic cell lines
x	Palaeontology and archaeology
x	Animals and other organisms
x	Human research participants
x	Clinical data
x	Dual use research of concern

Methods

n/a	Involved in the study
x	ChIP-seq
x	Flow cytometry
x	MRI-based neuroimaging
